# Gout Activity Score has predictive validity and is sensitive to change: results from the Nottingham Gout Treatment Trial (Phase II)

**DOI:** 10.1093/rheumatology/key446

**Published:** 2019-02-04

**Authors:** Sandra P Chinchilla, Michael Doherty, Abhishek Abhishek

**Affiliations:** 1 University of the Basque Country (UPV/EHU), Bilbao-Biscay, Spain; 2 Academic Rheumatology; 3 NIHR-BRC, University of Nottingham, Nottingham, UK

**Keywords:** gout, disease activity, quality of life

## Abstract

**Objectives:**

To examine the predictive validity of the Gout Activity Score (GAS), its correlation with the Gout Impact Scale (GIS) and their sensitivity to change.

**Methods:**

Data from a clinical trial in which participants with one or more gout flares in the previous year were recruited from primary care and randomized to nurse-led or continuing usual care were used in this study. GAS and GIS were calculated as described, with higher scores indicating worse disease activity and quality of life, respectively. The correlation between GAS and GIS was examined using Spearman’s correlation. Standardized response means (SRMs) were calculated to assess sensitivity to change. The association between GAS at baseline and the number of flares in the next 12 months was evaluated using Poisson regression. Data analyses were performed using STATA version 14, with *P*-values <0.05 being statistically significant.

**Results:**

There was low positive correlation between GAS and gout concern overall and unmet treatment need subscales of GIS (*r* = 0.34–0.45). Female sex associated independently with fewer gout flares, while increasing GAS, BMI and age associated independently with frequent flares. Of all the outcome measures examined, GAS was the most responsive to change (SRM 0.89 to −0.53). Of the GIS domains, the gout concern overall domain had the best sensitivity to change (SRM 1.06–0.01).

**Conclusion:**

GAS is sensitive to change, has predictive validity and correlates with relevant domains of GIS such as gout concern overall. Additional independent validation of GAS is required before it can be adopted in clinical practice.

**Rheumatology key messages**

- Gout activity score predicts future gout flares.
- Gout activity score is sensitive to change.
- Gout activity score correlates with some, but not all gout impact scale domains.

## Introduction

Gout is the most common inflammatory arthritis worldwide and is increasing in prevalence. It is estimated to affect 2–3% of people in the USA and Europe [1, 2]. The first disease-specific activity score for gout, the Gout Activity Score (GAS), was developed in 2016 and demonstrated low-level correlation with the HAQ and Patient Acceptable Symptom State and moderate correlation with pain [3, 4]. In a subsequent primary care–based study, it demonstrated negligible to low correlation with individual domains of the Gout Impact Scale (GIS), the only disease-specific quality of life (QoL) measure for gout [5]. The predictive validity of GAS and its sensitivity to change have not been examined. It is important to explore these properties of GAS to evaluate its validity as an outcome measure. Similarly, the sensitivity to change of GIS has not been examined in a clinical trial involving urate-lowering treatment (ULT), having previously been investigated in a longitudinal observational study, and in a trial of rilonacept or placebo for flare prophylaxis [6, 7]. 

Therefore the objectives of this study were to examine the correlation between GAS and GIS, examine the validity of GAS in predicting the number of gout flares and evaluate the responsiveness of GAS and GIS, using data from the Nottingham Gout Treatment Trial (phase II) [8, 9]. 

## Methods

### Data source

Data from the Nottingham Gout Treatment Trial (phase II) were used. The details of this study have been published elsewhere [8]. In brief, 517 community-derived adults with gout having one or more gout flares in the 12 months prior to recruitment were randomized 1:1 to either study arm and followed for 2 years. Patients in the usual care arm continued being treated by their general practitioner (GP), while those in the nurse-led arm followed a protocol that included patient education and engagement, addressing illness perceptions and a treat-to-target ULT strategy, reflecting recommended best practice according to rheumatology gout management guidelines [10, 11]. Research assessments at baseline, 1 year and 2 years included collection of demographic data, gout flares in the previous 12 months, gout activity questionnaire (GAQ 2.0) [12], medications, comorbidities, anthropometric measurements, tophus count and blood collection. The study was approved by the East Midlands Nottingham Research Ethics Committee (12/EM/0044) and registered with the National Clinical Trials Registry (www.clinicaltrials.gov, ref: NCT01477346).

### Calculation of outcome measures

GAS was calculated using the GAS_3-step-c_ formula [3]. This utilizes data on the self-reported number of attacks in the previous 12 months, serum urate, patient-reported visual analogue scale (VAS) of gout severity and the number of tophi. The subscales of GIS, specifically the gout concern overall, gout medication side effects, unmet gout treatment need, well-being during attack and gout concern during attack, were calculated as described by Hirsch *et al.* [12]. Higher scores for GAS and GIS indicate worse disease activity and QoL, respectively.

### Gout status change

As flares are an important patient-centred outcome, participants were classified into one of the four disease status change groups based on the number of self-reported flares in the 12 month period prior to study entry and the number of gout flares in the 12 months prior to the final 2 year research assessment visit. The disease state changes were

Flare free: No flares in the 12 month period preceding the 2 year research visit.

Better: At least one fewer flare in the 12 month period preceding the 2 year research visit compared with the 12 month period prior to study entry and not meeting criteria for flare free.

Same: An equal number of flares in the two time periods.

Worse: One or more flares in the 12 month period preceding the 2 year assessment than in the 12 month period prior to study entry.

### Statistical analysis

Mean (s.d.) and *n* (%) were used for descriptive purposes. As GIS and GAS were non-normally distributed, Spearman’s correlation test was used. The associations between baseline GAS and the number of flares in the next 12 months were examined using Poisson regression and were adjusted for age (tertiles); sex; BMI (tertiles); use of either NSAIDs, corticosteroids or colchicine at the baseline visit (yes/no) and the duration for which each participant was in the trial up to week 52. Crude and adjusted incidence rate ratios (IRRs) and 95% CIs were calculated to examine associations. As participants randomized to nurse-led care received up-titrated ULT, which may affect flare frequency, this analysis was restricted to participants in the usual care group alone *a priori*. A sensitivity analysis was performed, restricted to participants on stable dose ULT or not on any ULT.

Responsiveness to change was estimated using the standardized response mean (SRM). The SRM is calculated as a ratio of the mean change and s.d. of change scores from baseline and the end-of-study visit [13]. Data from all participants completing the 2 year randomized controlled trial and completing the GIS at the baseline and final study visit were included in the assessment of responsiveness to change. All data analysis was performed using STATA version 14 (StataCorp, College Station, TX, USA).

## Results

Data from 517 study participants, 262 receiving usual (GP-led) care and 255 receiving nurse-led care of gout, were included in this study. Briefly, there were 461 (89.2%) men, 203 (39.3%) on ULT, and 58 (11.2%) had one or more tophus at the baseline visit. Their mean age, BMI and serum urate was 62.89 years (s.d. 11.40), 29.79 kg/m^2^ (s.d. 5.07) and 7.41 mg/dl (s.d. 1.67), respectively. Their median disease duration and VAS pain were 9.54 years [interquartile range (IQR) 3.62–18.20] years and 4 mm (IQR 1–7), respectively.

There was low positive correlation between gout concern overall and the unmet treatment need subscales of GIS and GAS at all time points (Supplementary Table S1, available at *Rheumatology* online). Female sex associated with fewer gout flares [adjusted IRR (aIRR) 0.71 (95% CI 0.51, 1.00)], while increasing GAS quartiles [aIRR 1.34 (95% CI 1.23, 1.46)], BMI tertiles [aIRR 1.16 (95% CI 1.03, 1.30)] and age tertiles [aIRR 1.12 (95% CI 1.00, 1.26)] associated with frequent flares (Table 1). Participants taking anti-inflammatory drugs at the baseline visit had more flares. Twenty-seven people in the usual care arm commenced on ULT or changed its dose in the 12 months following the baseline visit. The association between increasing GAS quartiles and number of gout flares did not change when these participants were excluded in a sensitivity analysis [aIRR 1.20 (95% CI 0.86, 1.68), 2.08 (1.52, 2.83), 2.56 (1.87, 3.50) in the second, third and fourth GAS quartile with GAS in the first quartile as referent].

**Table 1:** Association between disease and demographic factors and GAS at baseline and number of flares during the next 12 months

Variable		IRR (95% CI)	*P*-value	aIRR (95% CI)	*P*-value
Gender	Male	1		1	
	Female	0.69 (0.50, 0.95)	0.024	0.71 (0.51, 1.00)	0.047
Age (years)	≤60.00	1		1	
	60.02–69.49	1.59 (1.26, 2.01)	<0.001	1.71 (1.35, 2.16)	<0.001
	≥69.60	1.06 (0.83, 1.36)	0.644	1.33 (1.03, 1.73)	0.030
BMI (kg/m^2^)	≤27.46	1		1	
	27.47–30.83	1.73 (1.39, 2.17)	<0.001	1.54 (1.23, 1.94)	<0.001
	≥30.84	1.34 (1.06, 1.70)	0.015	1.29 (1.01, 1.64)	0.042
GAS	≤3.46	1		1	
	3.47–4.33	1.78 (1.32, 2.42)	<0.001	1.61 (1.18, 2.18)	<0.001
	4.34–5.39	2.05 (1.53, 2.75)	<0.001	2.02 (1.50, 2.72)	<0.001
	≥5.40	2.81 (2.11, 3.74)	<0.001	2.61 (1.96, 3.49)	<0.001
Anti-inflammatory drugs	No	1		1	
	Yes	1.56 (1.26, 1.93)	<0.001	1.45 (1.15, 1.81)	0.001

Of all the outcome measures examined, GAS was the most responsive to change, with SRMs ranging from 0.89 in the flare free to −0.53 in those with worsening gout (Table 2). Of the GIS domains, the gout concern overall domain had the best sensitivity to change.

**Table 2:** Sensitivity to change of GAS and GIS

			GIS domains									
Gout status at year 2	GAS		Gout concern overall		Gout medication side effects		Unmet gout treatment need		Well-being during attack		Gout concern during attack	
	Mean **Δ**^a^ (s.d.**Δ**)^b^	SRM^c^	Mean **Δ** (s.d.**Δ**)	SRM	Mean **Δ** (s.d.**Δ**)	SRM	Mean **Δ** (s.d.**Δ**)	SRM	Mean **Δ** (s.d.**Δ**)	SRM	Mean **Δ** ( s.d.**Δ**)	SRM
Flare free (*n* = 282)	1.41 (1.59)	0.89	30.26 (28.51)	1.06	14.69 (28.60)	0.51	18.58 (25.54)	0.73	7.56 (21.51)	0.35	10.80 (23.01)	0.47
Better (*n* = 79)	1.35 (1.34)	1.01	22.24 (23.85)	0.93	12.00 (27.60)	0.43	21.38 (19.88)	1.08	10.15 (19.35)	0.52	3.65 (20.16)	0.18
Same (*n* = 50)	0.48 (1.63)	0.29	9.69 (27.24)	0.36	9.18 (28.62)	0.32	5.76 (30.18)	0.19	8.45 (22.32)	0.38	−0.94 (17.16)	−0.05
Worse (*n* = 18)	−0.51 (0.96)	−0.53	0.35 (28.76)	0.01	−2.08 (26.86)	−0.08	−2.31 (23.71)	−0.10	9.72 (20.41)	0.48	3.13 (17.84)	0.18

a MeanΔ (mean change) = [baseline score – score at year 2]/number of observations.

b s.d.Δ (s.d. of change) is the s.d. of the difference in the scores between baseline and year 2.

c SRM = meanΔ/s.d.Δ. Improvement is indicated by a positive change score and worsening by a negative change score.

## Discussion

This is the first study to examine the predictive validity and sensitivity to change of the GAS. It reports that increasing GAS associates with gout flares over a 12 month period, providing construct validity. GAS is the first composite disease activity measure for gout. It can be implemented easily in clinical practice and does not have any additional cost implications. The measurement and categorization of the DAS enables practitioners to communicate with patients clearly and increase treatment if necessary. Widespread use of such DASs has resulted in improved standards of care in other rheumatic conditions, such as RA. It is anticipated that the development and validation of a DAS for gout will improve the quality of care for gout.

In a previous study, gout patients meeting the preliminary definition of remission had a GAS ⩽2.78 [5]. Scire *et al.* [3] reported that a GAS <2.5 had the best ability to discriminate gout cases in remission. Taken together, these studies suggest that a GAS <2.5 is a reasonable long-term treatment target for gout. However, further research is required to identify the GAS cut-offs that represent moderate and high disease activity.

The GIS is the only disease-specific QoL instrument for gout. Our study demonstrates that the GAS has low positive correlation with the gout concern overall and unmet gout treatment need domains of the GIS, but poor to no correlation with the other three GIS domains. This is an expected finding, as the other three domains of the GIS are centred on side effects of treatment or QoL during flares. The magnitude of correlation between the GAS and GIS were comparable over a 2 year period and between intervention and control groups, suggesting that the relationship between the GAS and individual domains of the GIS is stable over time and between disease states.

There is no agreed definition of worsening of the clinical disease state in gout. We used an empirical definition of at least one more or one fewer gout flare in a 12 month period to indicate a clinical disease state change in gout based on feedback from patients and public involvement in research meetings in Nottingham, UK (AA personal communication). Using this definition, both the GAS and the gout concern overall and unmet treatment need domains of the GIS were sensitive to change. Numerically, the GAS had the greatest spread of SRMs and reflected disease worsening with a negative score. Thus it meets the truth and discrimination domains of the OMERACT filter 2.0 [14]. However, none of the outcome measures could differentiate between flare free and improved disease states of gout, and further refinement in outcome measures may be necessary.

This study reports that the GIS is more sensitive to change than previously reported. For example, the gout concern overall and unmet gout treatment need domains of the GIS had a sensitivity to change of 0.43 and 0.22, respectively, in previous studies [6, 7]. In this study, the mean change in the unmet gout treatment need domain of the GIS among people whose disease status remained unchanged was smaller than the minimally important differences for them reported previously (5.76 *vs* 6.88), whereas the mean change in the gout concern overall domain was only marginally higher compared with previous reports (9.69 *vs* 7.16), providing external validity to our findings [7]. We found an association between male sex, increasing BMI and frequent gout flares as reported previously [15, 16]. However, the observed association between age and gout flares is consistent with some but not all studies [15–18].

The strengths of this study include large sample size, community-based recruitment and standardized assessment of tophi. However, there are several caveats to this study. First, the number of gout flares in the 12 months prior to the baseline visit was self-reported, whereas the number of flares in the next 24 months was recorded prospectively. Furthermore, we did not utilize the recent definition of gout flares since the start of this study predated its publication [19].

In conclusion, the GAS is sensitive to change and has predictive validity. However, further research is required to define the GAS cut-offs that can be used to define states of high, moderate and low disease activity before it can be used in the clinic.

*Conflicts of interest:* A.A. has received speaking fees from Menarini and research grants from AstraZeneca and Oxford Immunotec. M.D. has received consulting fees and/or honoraria from AstraZeneca, Grunenthal, Mallinkrodt and Roche and research grants from AstraZeneca and Oxford Immunotec. S.P.C. declares no conflicts of interest.

*Funding:* This work was supported by Versus Arthritis (grant number 19703).

## Supplementary Material

key446_Supplementary_Data
