# Supplementary material for: Gout Activity Score has predictive validity and is sensitive to change: results from the Nottingham Gout Treatment Trial (Phase II)
Source: Rheumatology (Oxford). 2019 Feb 4;58(8):1378–82. doi: 10.1093/rheumatology/key446 (PMC6649838; doi:10.1093/rheumatology/key446)
Supplement: key446_Supplementary_Data [file key446_supplementary_data.docx]

Table S1: Correlation between GAS and individual domains of the GIS

| GIS | GAS | | | | | | | | | |
| --- | --- | --- | --- | --- | --- | --- | --- | --- | --- | --- |
|  | Baseline | | | 12 Months | | | 24 Months | | | |
|  | Both groups | Usual care | Nurse led care | Both groups | Usual care | Nurse led care | Both groups | Usual care | Nurse led care |  |
| Gout concern overall | 0.418^*^ | 0.404* | 0.428* | 0.427^*^ | 0.384* | 0.395* | 0.451^*^ | 0.434* | 0.353* |  |
| Gout medication side effect | 0.229^*^ | 0.182* | 0.275* | 0.233^*^ | 0.295* | 0.226* | 0.163^*^ | 0.188* | 0.160* |  |
| Unmet gout treatment need | 0.368^*^ | 0.341* | 0.383* | 0.427^*^ | 0.268* | 0.425* | 0.455^*^ | 0.362* | 0.362* |  |
| Well-being during attack | 0.269^*^ | 0.286* | 0.244* | 0.090 | 0.207* | 0.066 | -0.010 | 0.090 | -0.031 |  |
| Gout concern during attack | 0.304^*^ | 0.352* | 0.244* | 0.183^*^ | 0.277* | 0.190* | 0.191^*^ | 0.250* | 0.197* |  |

**p*<0.05
